# PNA-Based Graphene Oxide/Porous Silicon Hybrid Biosensor: Towards a Label-Free Optical Assay for Brugada Syndrome

**DOI:** 10.3390/nano10112233

**Published:** 2020-11-10

**Authors:** Rosalba Moretta, Monica Terracciano, Nicola Borbone, Giorgia Oliviero, Chiara Schiattarella, Gennaro Piccialli, Andrea Patrizia Falanga, Maria Marzano, Principia Dardano, Luca De Stefano, Ilaria Rea

**Affiliations:** 1 Institute of Applied Sciences and Intelligent Systems, National Research Council, 80131 Naples, Italy; rosalba.moretta@na.isasi.cnr.it (R.M.); principia.dardano@na.isasi.cnr.it (P.D.); ilaria.rea@na.isasi.cnr.it (I.R.); 2 Department of Pharmacy, University of Naples Federico II, 80131 Naples, Italy; nicola.borbone@unina.it (N.B.); gennaro.piccialli@unina.it (G.P.); 3 Department of Molecular Medicine and Medical Biotechnologies, University of Naples Federico II, 80131 Naples, Italy; giorgia.oliviero@unina.it (G.O.); andreapatrizia.falanga@unina.it (A.P.F.); 4 Department of Physics “E. Pancini”, University of Naples Federico II, 80126 Naples, Italy; ch.schiattarella@gmail.com; 5 Institute of Crystallography, National Research Council, 70126 Bari, Italy; maria.marzano@unina.it

**Keywords:** peptide nucleic acid-PNA, porous silicon, graphene oxide, label-free optical biosensor, hybrid device, Brugada syndrome

## Abstract

Peptide nucleic acid (PNA) is a synthetic DNA mimic that outperforms the properties of traditional oligonucleotides (ONs). On account of its outstanding features, such as remarkable binding affinity towards complementary DNA or RNA as well as high thermal and chemical stability, PNA has been proposed as a valuable alternative to the ON probe in gene-sensor design. In this study, a hybrid transducer made-up of graphene oxide (GO) nano-sheets covalently grafted onto a porous silicon (PSi) matrix has been investigated for the early detection of a genetic cardiac disorder, the Brugada syndrome (BS). A functionalization strategy towards the realization of a potential PNA-based device is described. A PNA, able to detect the SCN5A gene associated with the BS, has been properly synthesized and used as a bioprobe for the realization of a proof-of-concept label-free optical PNA-biosensor. PSi reflectance and GO photoluminescence signals were simultaneously exploited for the monitoring of the device functionalization and response.

## 1. Introduction

Nucleic acid analogues have been recognized as powerful tools in DNA biosensor design as functional bioprobes. Beyond the most well-known DNA and RNA analogues (e.g., negatively charged phosphorothioate oligonucleotide-PTO; locked nucleic acid-LNA), the uncharged peptide nucleic acid (PNA) is the most used in biosensing applications. PNA is a synthetic DNA analogue whose backbone is formed of N–(2-aminoethyl)glycine motifs linked by peptide bonds [1]. This molecule is considered a better probe then traditional oligonucleotide (ON) sequence in DNA and RNA targeting. The hybridization to a complementary sequence obeys the hydrogen bonding rule (Watson–Crick), and the uncharged backbone of PNA allows the formation of more stable PNA/DNA and PNA/RNA complexes with respect to the corresponding DNA/DNA or DNA/RNA systems [2,3]. The neutral charge nature of the amide backbone also enables the PNA hybridization to the target sequence on exposure to a low-salt environment. In this case, the salt positive ions are not necessary for neutralizing the inter-strand repulsion that hampers the duplex formation between two negatively charged ONs [4,5,6,7,8]. Moreover, the PNA is chemically and thermally stable in those environments in which the nucleic acids would undergo a complete degradation [9]. The PNA is also a powerful probe to detect single-base mismatches in DNA and thus a suitable tool for the development of a biosensor (i.e., gene-sensor) for the diagnosis of point mutation-related genetic diseases, such as the Brugada syndrome (BS). 

The BS is a cardiac disease characterized by some typical electrocardiogram (ECG) alterations (i.e., polymorphic ventricular tachycardia, ventricular fibrillation), associated with a high risk of sudden cardiac death, which affects mainly the young people without any morphological heart malformations [10]. The syndrome is classified as a channelopathy, caused by an electrical dysfunction of the cardiac ion channels which participate in the action potential [11]. This disorder has a genetic basis with the autosomal dominant transmission, and numerous mutations have been identified in the genes encoding cardiac ion channel subunits as well as in the genes regulating their activity [12,13]. Most of the mutations related to this syndrome have been found in the SCN5A gene, encoding the α subunit of the cardiac sodium channel (locus 3p21, 28 exons) [14]. Currently, the BS diagnosis is based on the ECG, which identifies some characteristic electrocardiographic patterns, and also on expensive as well as time-consuming laboratory genetic tests [15]. Unfortunately, in most cases, BS is not diagnosed until a fatal event occurs.

Advanced biosensors could be the most effective approach to solve some of the issues concerning the sensitive, fast, and cost-effective monitoring of BS [16,17,18,19,20]. In this frame, nanostructured materials have been massively introduced in biosensing applications due to their novel and unique physicochemical properties [21,22]. 

In this study, two inorganic nanostructured materials, graphene oxide (GO) and porous silicon (PSi), were combined to develop an optical label-free PNA biosensor as a proof-of-concept device for the early detection of the BS. 

The GO is a chemically modified graphene, characterized by different oxygen functional groups (i.e., hydroxyl, epoxy, carboxyl, and carbonyl groups) on its basal plane and edges. The GO is thus water-soluble and exposes reactive sites for chemical functionalization [23,24,25,26,27,28]. Although the GO exhibits a wide photoluminescence (PL) emission from 500 to 800 nm on exposure to near UV radiation, its PL signal is normally weak because of the oxygen-containing functional groups favouring non-radiative recombination between their electrons and holes of the *sp2* clusters [29]. 

The PSi, obtained by the electrochemical dissolution of crystalline silicon in hydrofluoric acid (HF) based solution, is one of the most used nanostructured materials in biosensing, due to its unique features [20,30,31,32,33,34]. 

In our recent papers, GO nanosheets were electrostatically adsorbed on an amino-modified PSi multilayer and showed an enhanced as well as modulated PL signal, opening the way for the realization of a hybrid transducer with advanced optical properties [35,36]. The working stability of such a device has been improved by covalently binding the GO to the PSi surface, using a proper chemical procedure. The developed hybrid transducer has been covalently bound to a model bioprobe protein A (PrA) from *Staphylococcus aureus*, demonstrating the potentiality to develop a powerful biosensor in which PSi reflectance and GO photoluminescence represent the operating mechanisms [37]. In this work, a specific PNA bioprobe was covalently grafted to the hybrid optical GO/PSi transducer for the realization of PNA hybridization biosensor. The PNA bioprobe has been properly designed and synthesized by solid-phase strategy to detect a DNA target sequence related to the BS monitoring. The PNA-DNA selective recognition through heteroduplexes formation has been investigated in solution by using Circular Dichroism (CD), CD melting and non-denaturing Polyacrylamide Gel Electrophoresis (PAGE). The GO/PSi PNA-biosensor has been investigated by spectroscopic reflectometry, steady-state photoluminescence, scanning electron microscopy (SEM), atomic force microscopy (AFM), and fluorescence confocal microscopy. 

## 2. Materials and Methods

### 2.1. Chemicals

Undecylenic acid (UDA), 2-(N-morpholino)ethanesulfonic acid (MES) hydrate, hydrofluoric acid (HF), *N*-(3-dimethylaminopropyl)-*N*′-ethylcarbodiimide hydrochloride (EDC), *N*-hydroxysuccinimide (NHS), tetrahydrofuran (THF), dimethyl sulfoxide (DMSO), chloroform, tert-butyloxycarbonyl-NH-PEG-Amine (BOC-NH-PEG-NH2), trifluoroacetic acid (TFA) were purchased from Sigma Aldrich (St. Louis, MO, USA). Graphene oxide was purchased from Biotool.com (Houston, TX, USA) as a batch of 2 mg mL^−1^ in water with a nominal sheets size between 50 and 200 nm. Phosphoramidites for ONs syntheses were purchased from Glen Research (Sterling, VA, USA). PNA monomers were purchased from Link technologies (Bellshill, Lanarkshire, UK). Fmoc-L-Lys(MMT)-OH was purchased from Iris Biotech GmbH (Marktredwitz, Germany). All solvents for the syntheses of DNA and PNA molecules and MBHA resin (1% divinylbenzene, 200–400 mesh, 0.5 mmol/g loading) were purchased from Sigma-Aldrich (Saint Louis, MO, USA). The solid-phase reactions were carried out using ISOLUTE^®^ single fritted reservoirs (SG), 3 mL 20 µm PE (polyethylene), provided with tube caps, and luer tip caps (Biotage, Uppsala, Sweden) which were shaken in a Multi-reax vibrating shaker (Heidolph, Schwabach, Germany).

### 2.2. Preparation of Graphene Oxide and DLS Characterizations

Graphene oxide (GO), 1.5 mg mL^−1^, was sonicated for 1 h in ice, at 50% of available power amplitude (Bandelin Sonopuls UWmini20 with MS72/MS73 microtip). The GO dispersion was centrifuged for 90 min at 21,380× *g* (Scilogex SCI24R High Speed Refrigerated Micro-Centrifuge) and the pellet was dissolved in DMSO. The hydrodynamic diameter (Z-average) of the obtained GO sheets was measured using a Zetasizer Nano ZS instrument (Malvern Instruments Ltd., Worcestershire, UK) equipped with a He-Ne laser (λ_em_ = 633 nm), at a fixed scattering angle of 173°, T = 25 °C. 

### 2.3. Porous Silicon Fabrication and Hydrosilylation Process

The single-layer PSi structure was obtained by electrochemical etching of n-type crystalline silicon (0.01–0.02 Ω cm resistivity, <100> orientation, 500 µm thickness) in 200 mL HF (5% in weight)/ethanol solution at room temperature (RT). The native oxide layer on the silicon substrate was removed by immersion in 10 mL HF (1% in weight)/ethanol solution for a few mins before the etching process. A PSi monolayer with porosity 61% (n_PSi_ = 1.83 at λ = 1.2 µm), thickness (L) 2.1 µm and pore size distribution varying from 50 to 250 nm was fabricated using a current density of 20 mA cm^−2^ for 90 s (refractive index and porosity have been obtained by spectroscopic variable angle ellipsometry, data not shown here). The fresh-made PSi was placed in a Schlenk tube containing 2 mL of deoxygenate neat UDA (99% *v*/*v*). The reaction was conducted at 110 °C for 18 h in argon. The hydrosilylated-PSi was extensively washed in chloroform and THF [38].

### 2.4. PEGylation of PSi Layer and Covalent Grafting of GO

The carboxyl acid groups of UDA were activated by EDC/NHS (0.005 M in 0.1 M MES buffer, pH 5) for 90 min at RT. Afterwards, a solution containing BOC-NH-PEG-NH_2_ (0.004 M, overnight at 4 °C) was used to completely recover the PSi chip. In addition, to remove the excess of reagent, the sample was rinsed in deionized water (three washes for 5 min). Furthermore, 5 mL of TFA solution (95% *v*/*v*, 90 min, RT) was used to deprotect the amino group of the PEG molecule. The PSi chip was washed with deionized water (three washes for 5 min). The sonicated GO was activated by EDC/NHS (0.029:0.020 M in DMSO, overnight, RT) and added to the PSi chip, in order to form the covalent bond. Then, the sample was rinsed three times in deionized water for 5 min to remove the unreacted GO.

### 2.5. DNA Synthesis and Analysis

The sequences of DNA-FAM, DNA and DNA polyT-FAM (Table 1) were obtained by solid-phase β-cyanoethyl phosphoramidite chemistry using a Perseptive Biosystem Expedite 8909 automated DNA synthesizer. The syntheses were carried out on a CPG Universal Support (35 mg, 1.4 µmol) using 1 µmol scale standard protocol, with the DMT-OFF option. For DNA-FAM and DNA polyT-FAM syntheses, 6-[(3′,6′-dipivaloylfluoresceinyl)-carboxamido]-hexyl-1-*O*-[(2-cyanoethyl)-(*N,N*-diisopropyl)]-phosphoramidite (FAM) was introduced in the last step of the synthesis of the oligonucleotides. The oligomers were removed from the support and deprotected by concentrated aqueous ammonia at 55 °C for 12 h. The filtrates and washings were concentrated under reduced pressure, redissolved in H_2_O, analyzed and purified by HPLC by Jasco (Easton, MD, USA) PU-2089 on a Nucleogen anion exchange column (Macherey-Nagel, 1000-8/46) using a linear gradient from 100% of buffer A (20 mM NaH_2_PO_4_ aqueous solution, pH 7.0, containing 20% (*v*/*v*) CH_3_CN) to 100% of buffer B (1 M NaCl, 20 mM NaH_2_PO_4_ aqueous solution, pH 7.0, containing 20% (*v*/*v*) CH_3_CN) in 30 min and the flow rate of 1.2 mL min^−1^. The ONs were collected and successively desalted by Sep-Pak cartridges (C18) and their concentrations were determined spectrophotometrically at λ = 260 nm and 90 °C, using the molar extinction coefficient ε = 217.6 for cm^−1^mM^−1^ for both DNA FAM and DNA, and 95.3 cm^−1^mM^−1^ for DNA polyT-FAM, determined using the Sigma-Aldrich OligoEvaluatorTM web tool (www.oligoevaluator.com).

### 2.6. PNA Synthesis, Purification, and Analysis

PNA sequence (Table 1) was synthesized using the 9-fluorenylmethoxycarbonyl (Fmoc) solid-phase strategy, following the protocol reported elsewhere [6]. The raw product was purified by semipreparative HPLC analyses by Jasco (Easton, MD, USA) Plus pump equipped with a Jasco UV-2075 Plus UV detector using a 10 × 250 mm C-18 reverse-phase column (particle size 5 µm) (Merck Millipore Billerica, MA, USA) eluted with a linear gradient of CH_3_CN containing 0.1% (*v*/*v*) TFA in H_2_O containing 0.1% (*v*/*v*) TFA (from 0 to 100% in 45 min, flow 1.2 mL min^−1^). The purified PNA was lyophilized using a SCANVAC CoolSafe 55 freeze-dryer (ScanLaf A/S, Lynge, Denmark) by setting the freezing-temperature at –65 °C, vacuum pressure at 0.01 mbar, and heating temperature at +100 °C. After the lyophilization process, the sample was dissolved in pure water estimating its concentration by UV analyses by the Jasco V-530 spectrophotometer (λ = 220–310 nm, 400 nm min^−1^ scanning speed, 2.0 nm bandwidth; ε = 116.4 cm^−1^mM^−1^ for PNA. The product was characterized by ESI-MS Applied Biosystems 4000 QTRAP mass spectrometer. PNA: ESI-MS (m/z) calcd. for [M + 2H]^2+^ 1807.74, found 1807.7, calcd. for [M + 3H]^3+^ 1205.49, found 1205.5; calcd. for [M + 4H]^4+^ 904.37, found 904.4 (Figure S1).

### 2.7. Preparation of DNA/PNA Samples

DNA-FAM, DNA-FAM/PNA (1:0.8 ratio), DNA, DNA/PNA complex (1:0.8 ratio) and PNA samples were prepared in 100 mM phosphate-buffered saline (PBS), pH = 7.0. For DNA-FAM and DNA preparation, 10 nmol of each ON were lyophilized using a SCANVAC CoolSafe™ freeze-dryer and dissolved in 500 μL of 100 mM PBS buffer. DNA FAM/PNA and DNA/PNA mixtures were obtained at 1:0.8 (ON/PNA) ratio by mixing 10 nmol of lyophilized ON with 8 nmol of PNA solution 1 mM (8 µL) and diluting to 500 μL with 100 mM PBS buffer. Resulting solutions were heated at 90 °C for 10 min, equilibrated at 4 °C overnight, and finally used for CD and PAGE analysis.

### 2.8. Circular Dichroism (CD) and CD Melting

CD spectra analysis was carried out by Jasco 1500 spectropolarimeter equipped with a Jasco PTC-348-WI temperature controller in the 200–320 nm range at 5 °C, using 0.1 cm path-length cuvette in 100 mM PBS at the concentration of 20 µM. All CD spectra were acquired at 200 nm min^−1^ scan rate, 4 s response time, 2 nm bandwidth, and averaged over five scans. Buffer baseline subtraction and normalization was carried out for each spectrum thus having zero ellipticity at 360. Thermal denaturation experiments were performed in the temperature range 5–90 °C by monitoring the CD values at 269 nm for DNA FAM/PNA and 268 nm for DNA/PNA heteroduplexes at a heating rate of 1 °C min^−1^.

### 2.9. Non-Denaturing Polyacrylamide Gel Electrophoresis (PAGE)

PAGE was performed using 18% polyacrylamide gels, which were run in 1 × Tris-Borate-EDTA (TBE) buffer supplemented with 30 mM KCl, pH 7.0 for 1 h at 4 °C. The samples were loaded at 20 µM ON concentration. Before gel loading, 2 µL of each sample was added to 8 µL of loading buffer (glycerol/1 × TBE + 30 mM KCl 1:9). Electrophoresis was carried out at a constant voltage of 120 V at 5 °C. The gel was stained with Sybr green (Sigma-Aldrich) and visualized by Bio-Rad Laboratories Gel Doc XR apparatus.

### 2.10. Covalent Grafting of PNA on PSi Layer and DNA Hybridization

The GO/PSi device was exposed to 100 µM of PNA in presence of EDC/NHS (0.040:0,016 M in MES buffer, overnight, RT) and washed in deionized water.

Complementary (DNA, AGGAGAGCACCGAGCCCCTGAG) and non-complementary (DNA polyT, CCTTTTTTTTTT) DNA sequences with and without FAM chromophore (Table 1) were incubated on the chip for two hours. The sample was gently rinsed in deionized water to remove the unhybridized target.

### 2.11. Spectroscopic Reflectometry

The optical setup to acquire the reflectivity spectra of PSi monolayer is made up of a white light source and the Ando AQ6315B optical spectra analyser (Yokohama Italia S.r.l, Nova Milanese, Italy), both connected to a Y reflection probe (Avantes, Apeldoorn, The Netherlands), through which the light is conveyed and subsequently collected to/from the sample at normal incidence. The acquisition of the optical spectra was performed from 600 to 1600 nm with a sampling wavelength of 1 nm. The reflectivity spectra are the result of the average of three independent measurements.

### 2.12. Steady-State Photoluminescence

A continuous wave KIMMON He-Cd laser (KIMMON KOHA, Tokyo, Japan) at wavelength of 442 nm was used to excite the steady-state photoluminescence from the samples. The PL spectra were acquired by an optical fiber directly connected to a SpectraPro 300i spectrometer (Teledyne Princeton Instruments, Trenton, NJ, USA), and a PIXIS 100F CCD camera (Teledyne Princeton Instruments) equipped with a Peltier cooling system. The laser line signal was cut from the PL spectra by using a long pass filter (λ_cut-on_ = 458 nm), applied to the monochromator inlet.

### 2.13. Laser Scanning Confocal Microscopy

Fluorescence images were obtained by inverted confocal microscopy on a fully automated Nikon A1R instrument (Nikon Instruments Europe BV, Amsterdam, The Netherlands), collected and analyzed by NIS element software.

### 2.14. Scanning Electron Microscopy

SEM characterization was performed by a Carl Zeiss NTS GmbH 1500 Raith Field Emission Scanning Electron Microscope (Carl Zeiss, Oberkochen, Germany), equipped with an InLens detector, by setting 5 kV voltage and 30 µm aperture. The side view of the samples was obtained by tilting them of 90°.

### 2.15. Atomic Force Microscopy

An XE-100 atomic force microscope (Park Systems Europe GmbH, Mannheim, Germany) was used for imaging of PSi samples. Surface imaging was obtained in Non-Contact Mode (NCM), using silicon/aluminum coated cantilevers (SSS-NCHR 10M; Park Systems Europe GmbH) 125 μm long with a resonance frequency of 204 to 397 kHz nominal force constant of 42 N m^−1^ and a typical tip radius 2 nm (<5 nm max). The scan frequency was typically 0.35 Hz per line. AFM images were analyzed by the program XEI 1.8.1.build214 (Park Systems Europe GmbH).

## 3. Results and Discussion

The GO is a two-dimensional material obtained by graphite oxidation and can be considered the hydrophilic counterpart of the graphene, due to the oxygenated functional groups [23]. The oxygen-containing functional groups on the GO can be also used for binding other molecules on its surface or for graft the nano-sheets on different substrates [39,40]. The realization of hybrid GO/PSi transducers was obtained by the covalent grafting of GO carboxyl acid groups (−COOH) to an opportunely modified macroporous silicon matrix. A proper sonication process was mandatory to get GO nanosheets small enough to penetrate into the porous matrix [37]. After sonication, the size distribution of the GO was investigated by DLS analysis, revealing the presence of main peak corresponding to 25 ± 5 nm size population.

**Figure 1:**
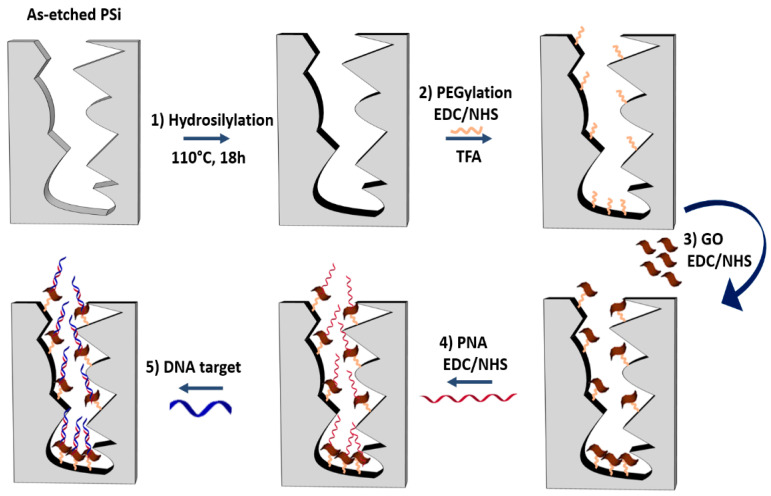
Functionalization scheme of GO/PSi hybrid device with the PNA probe and hybridization with the DNA target. Reaction 1: hydrosilylation of PSi by UDA (18 h at 110 °C). Reaction 2: PEGylation of PSi by EDC/NHS chemistry and deprotection of NH-BOC group by TFA treatment. Reaction 3: GO conjugation to PEGylated-PSi by EDC/NHS. Reaction 4: immobilization of the PNA by a covalent chemistry. Reaction 5: DNA target recognition by hybridization.

Since PSi optical biosensors are strongly limited by the oxidation and degradation of its porous matrix under ambient conditions (i.e., on exposure to air or aqueous solutions) [30,38,41], a passivation process was required before the GO infiltration. A schematic diagram of the PSi surface functionalization is reported in Figure 1.

The passivation of the PSi surface was performed through the hydrosilylation with UDA which replaced the silicon hydride bonds (Si−H) with silicon alkyls (Si−C), conferring a greater chemical stability to the material as well as functional carboxyl groups (Figure 1, reaction 1) [38]. A homofunctional amino-terminal PEG molecule was used as a cross-linker to covalently bind the GO onto the PSi surface by standard EDC/NHS chemistry (Figure 1, reaction 2). The same chemistry was used for grafting the GO nanosheets to the obtained amino-terminal PSi after the deprotection of −NH_2_ groups of the PEG by TFA treatment (Figure 1, reaction 3) [42]. The morphology of the PSi before and after the GO immobilization was investigated by SEM imaging. Top and side views of samples clearly showed that the GO nanosheets covered the PSi surface without blocking the pores (Figure S2A,B).

The BS is a genetic cardiac disorder identified by a typical heart arrhythmia due to voltage-gated sodium channel (NaV1.5) dysfunction. The charge reversal mutant E1784K is the most common mutation based on a guanine to an adenine substitution at position 5350 in SCN5A gene expressed in the C-terminal domain of NaV1.5 [43]. The PNA molecule complementary to a DNA sequence, corresponding to a tract of the mRNA codifying for the α subunit of the cardiac sodium channel, has been selected through bioinformatic tools using the online databases: UniProt-Ensemble and NCBI gene database [44,45].

The selective interaction between the synthetized PNA and the DNA target, and the stability of the resulting heteroduplexes were assessed in solution by using CD, CD melting, and PAGE analyses. A CD profile of DNA/PNA at 1:0.8 ratio (with and without FAM chromophore) resulted differently from those of free DNA and PNA, as well as of their arithmetic sum, thus confirming the heteroduplex formation (Figure S3 panels A and B, respectively).

**Figure 2:**
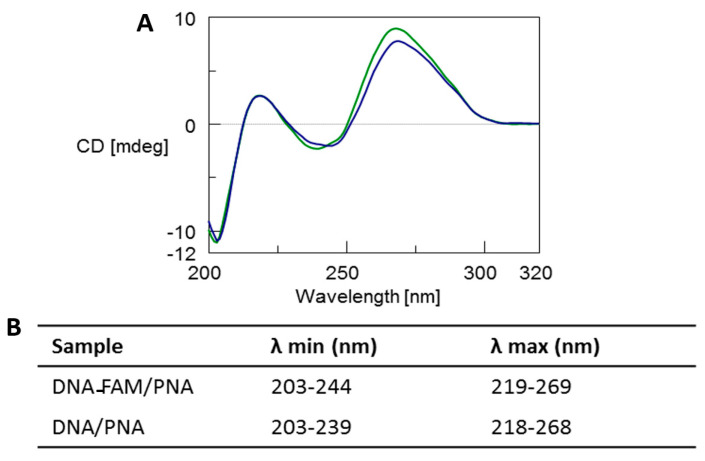
(**A**) Overlapped CD spectra of DNA-FAM/PNA (blue line) and DNA/PNA (green line) mixtures after annealing in 100 mM PBS at pH 7.0. (**B**) λ values of CD minima and maxima.

The CD profile of both the DNA-FAM/PNA and DNA/PNA complexes (Figure 2, blue and green line, respectively) showed the typical spectrum of antiparallel heteroduplexes characterized by two CD maxima around 260 nm and 220 nm and two minima around 240 nm and 200 nm, thus confirming the formation of the heteroduplexes [3,5,6,46]. Moreover, a slight reduction of the positive dichroic signals at 269 nm observed for the DNA-FAM/PNA in comparison with DNA/PNA profile could be ascribed to the presence of the FAM chromophore. CD melting analysis proved that the heteroduplex DNA-FAM/PNA showed a lower stability (Tm = 44 °C) compared to the complex without the FAM label (Tm = 50 °C) (Figure S4). However, the presence of FAM did not influence the shape of the CD profile of the heteroduplex.

**Figure 3:**
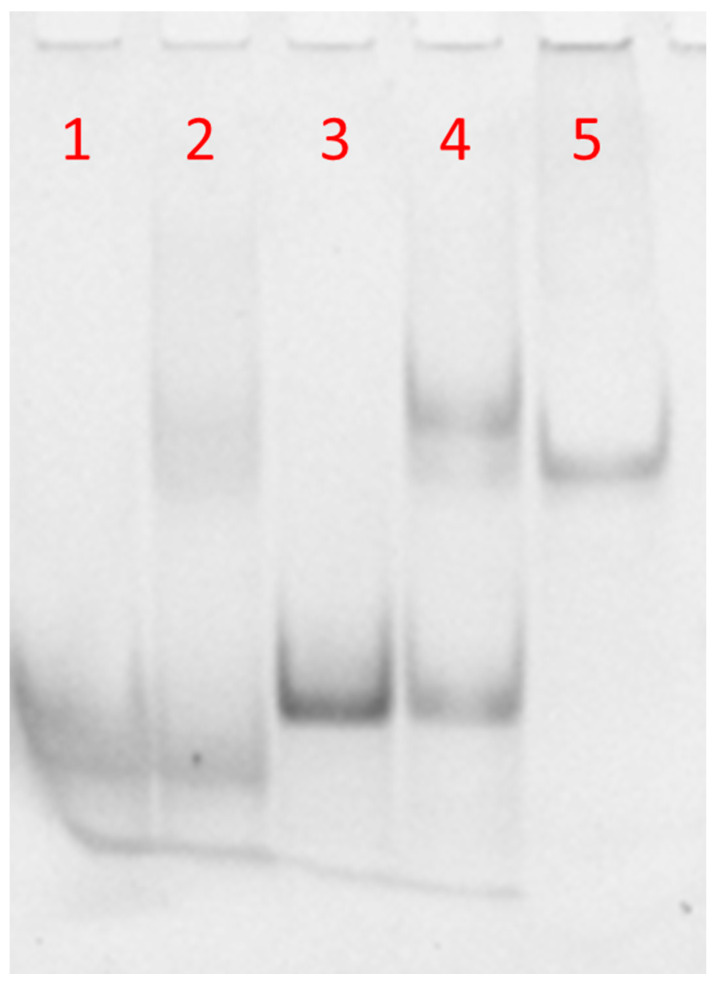
PAGE of DNA-FAM or DNA annealed with PNA in 100 mM PBS solution. Lane 1: DNA alone; lane 2: DNA/PNA mixture; lane 3: DNA FAM alone; lane 4: DNA FAM/PNA mixture, lane 5: 23-bp duplex DNA marker.

The molecular size of the synthetized complexes was examined by PAGE analysis in 100 mM PBS (Figure 3). In the case of free DNA and DNA-FAM, one main band corresponding to the single strand was observed (Figure 3, lanes 1 and 3, respectively). The PAGE mobility of the samples obtained after annealing DNA or DNA-FAM with PNA (1:0.8 ratio) showed a single strand band due to the DNA excess compared to PNA (Figure 3, lines 2 and 4, respectively). Moreover, the appearance of a new clear band migrating slower than the single strand, due to DNA or DNA-FAM/PNA heteroduplex, had almost the same mobility of a 23-bp reference duplex DNA (lane 5) [5,6]. The higher intensity of the bands in lanes 3 and 4 with respect to lanes 1 and 2 was ascribed to the presence of the FAM-labelled DNA in place of the free FAM-DNA. These results demonstrated that the hybridization between PNA and the DNA was not dependent on FAM functionalization. Several hybridization studies in solution have demonstrated that PNA/complementary ON recognition takes place by Watson–Crick base pairing, that is, A-T and G-C base pairing [3,46,47,48,49]. Therefore, no specific recognition could occur between the synthesized PNA and the non-complementary DNA polyT sequence in solution.

**Figure 4:**
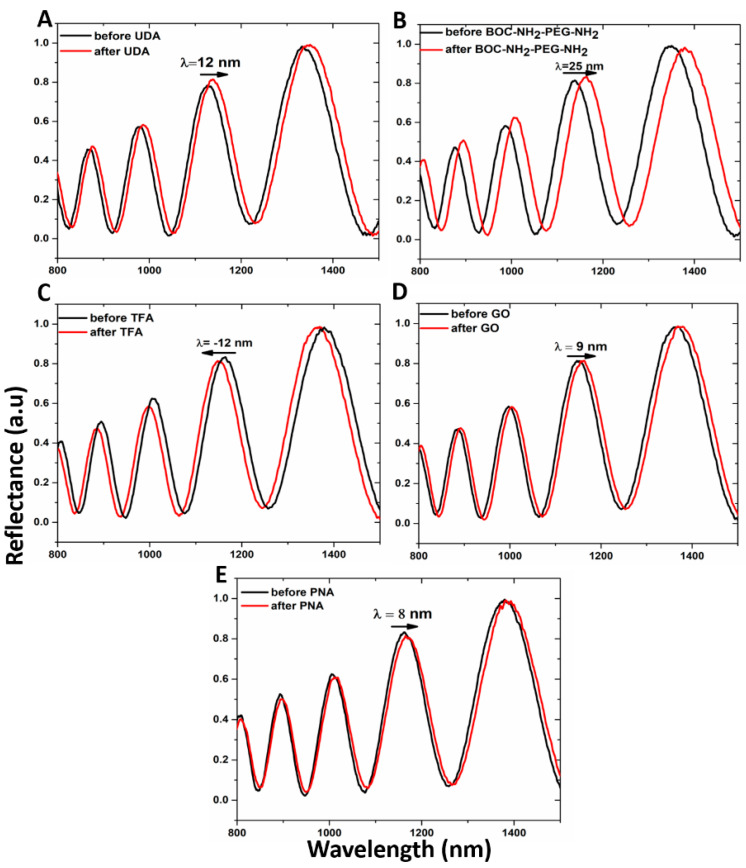
Reflectivity spectra of PSi (**A**) before (black line) and after (red line) UDA passivation, (**B**) before (black line) and after (red line) PEGylation with BOC-NH_2_-PEG-NH_2_, (**C**) before (black line) and after (red line) deprotection of amino group by TFA treatment, (**D**) before (black line) and after (red line) GO infiltration, (**E**) before (black line) and after (red line) PNA immobilization.

**Figure 5:**
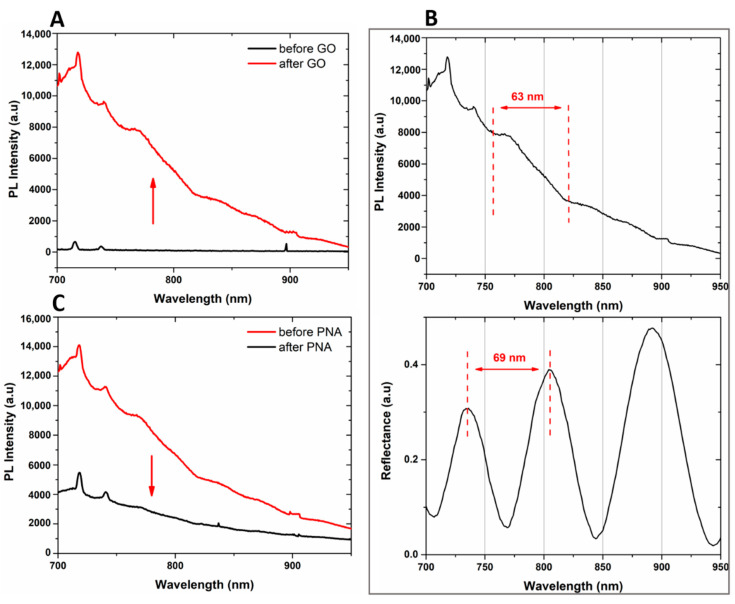
(**A**) photoluminescence spectra of PSi before (black line) and after (red line) GO infiltration at an excitation wavelength of 442 nm. (**B**) Comparison between PL spectrum (upper graph) and reflectivity spectrum (lower graph) of GO/PSi hybrid device. (**C**) PL spectra of PSi before (red line) and after (black line) PNA immobilization, at an excitation wavelength of 442 nm.

Both the passivation process of the hybrid GO/PSi device and the ONs interaction, after PNA grafting on the hybrid composite surface, were characterized by optical measurements. The normal incidence reflectivity spectra after each step of the functionalization are reported in Figure 4. Both the hydrosilylation and PEGylation processes caused the reflectivity spectra red-shifts of 12 and 25 nm, respectively (Figure 4A,B). This effect clearly highlighted the formation of organic layers on the pore walls, which increased the average refractive indexes of the PSi matrix. On the contrary, the deprotection of −NH_2_ groups by TFA treatment was confirmed by –12 nm of blue-shift of the spectrum due to the removal of the BOC protector group (Figure 4C). Material infiltration inside the pores caused the red-shift of 9 nm after the GO grafting, confirming the designed PSi functionalization (Figure 4D). Finally, the successful immobilization of PNA on the PSi surface induced a further spectrum shift of 8 nm (Figure 4E). PL measurements, by using an excitation wavelength of 442 nm, were executed to evaluate the infiltration of GO inside the porous matrix. As shown in Figure 5A, while no PL signal was detected in the case of bare PSi, the infiltration of GO into the PSi matrix was demonstrated by the wavelength modulation of the PL signal, clearly confirming the GO infiltration into the PSi matrix.

A comparison between the PL and reflectivity spectra of GO infiltrated in the PSi matrix is shown in Figure 5B. The PL signal could be explained by considering the Fabry–Perot interferometer theory. The emission wavelengths, denoted as λ_em,_ of GO into PSi, which fulfill the relationship L = m(λ_em_/2*n_PSi_*), where L is the thickness of the PSi monolayer and *m* an integer, could interfere constructively showing maxima in the photoluminescence spectrum of the hybrid device. The interval between two sequential PL maxima was 63 nm, in good agreement with the measured free spectral range of the designed hybrid device (GO/PSi), equal to 69 nm [35].

The bioprobe immobilization on the GO/PSi hybrid surface was confirmed by the quenching of GO PL, shown in Figure 5C. The oxygen-functionalities were the defects of the two-dimensional surface on which the emission properties of the material depend. The binding of PNA to carboxyl groups of GO nanosheets reduced the available radiative emission centers and thus also the PL signal [50].

The PNA-GO/PSi device was then incubated with complementary (DNA) and non-complementary (DNA polyT) sequences and this molecular interaction was analyzed by reflectance spectroscopy (Figure 1, reaction 5).

**Figure 6:**
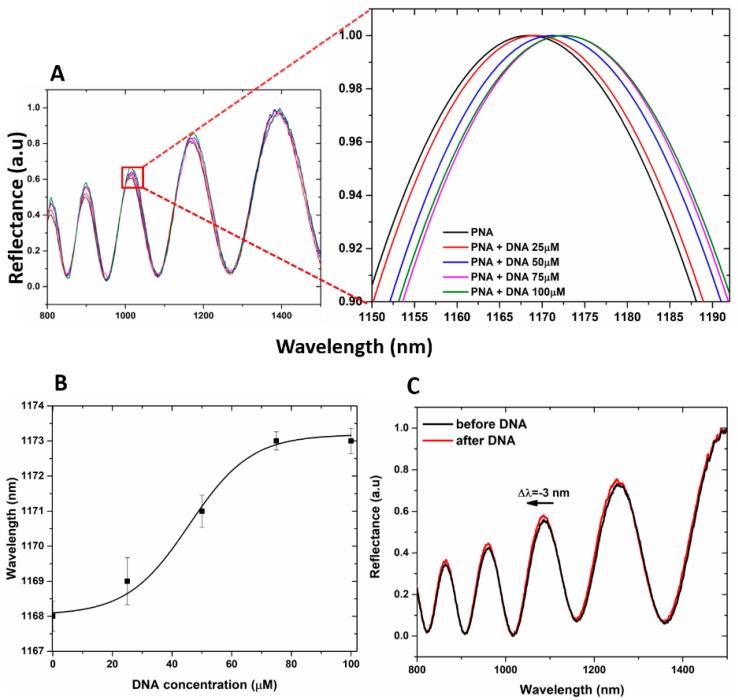
(**A**) reflectivity spectra of PNA-GO/PSi device after the DNA incubation at pH 5. (**B**) dose–response curve as a function of the DNA concentration (pH 5). Experimental data (black squares) were fitted by using OriginLab^TM^ Dose–response nonlinear curve fit. (**C**) reflectivity spectra of PNA-GO/PSi device after the DNA incubation at pH 7.5.

It is well known that the accumulation of negative surface charges on the PSi matrix might lead to the corrosion of the PSi layer due to the sum of the deprotonated free −COOH groups of GO and those of the polyanionic DNA target [51]. This is the reason why the hybridization experiments with DNA at increasing concentration (25, 50, 75, and 100 µM) on hybrid GO/PSi device were made in aqueous solution both at neutral and mild acidic pH conditions (7.2 and 5.0, respectively). In Figure 6A, the reflectivity spectra of PNA-GO/PSi hybrid device after incubation in DNA aqueous solution at pH 5.0 showed net red shift variations (1, 3, 5, and 5 nm, respectively) at increasing DNA concentrations (25, 50, 75 and 100 µM), thus demonstrating a matter accumulation and not any corrosion. The saturation was reached at target concentration of 100 µM. The dose–response curve as a function of DNA concentration is shown in Figure 6B. According to the IUPAC definition, the limit of detection (LOD) is calculated as three times the standard deviation of the blank signal divided by the sensitivity, 3σ/S. In our case, the blank is the sample at 0 µM (the sample functionalized with PNA strand). The corresponding LOD was 25 ± 2 µM, where σ (0.58 nm) is the standard deviation of the measured signal at 0 µM and S (0.07 ± 0.01 nm µM^−1^) is the sensor sensitivity, calculated in the linear range (between 0 and 75 µM, data not shown here) of the dose–response curve (R^2^ = 0.98). PNA-GO/PSi hybrid device after incubation in DNA aqueous solution at pH 7.2 showed net blue shift variations (data not shown) at increasing DNA concentrations (25, 50, 75 and 100 µM). Figure 6C reports representative spectra after the hybridization with the DNA (100 µM) in a neutral condition and a clear blue-shift of −3 nm confirmed the PSi matrix corrosion.

**Figure 7:**
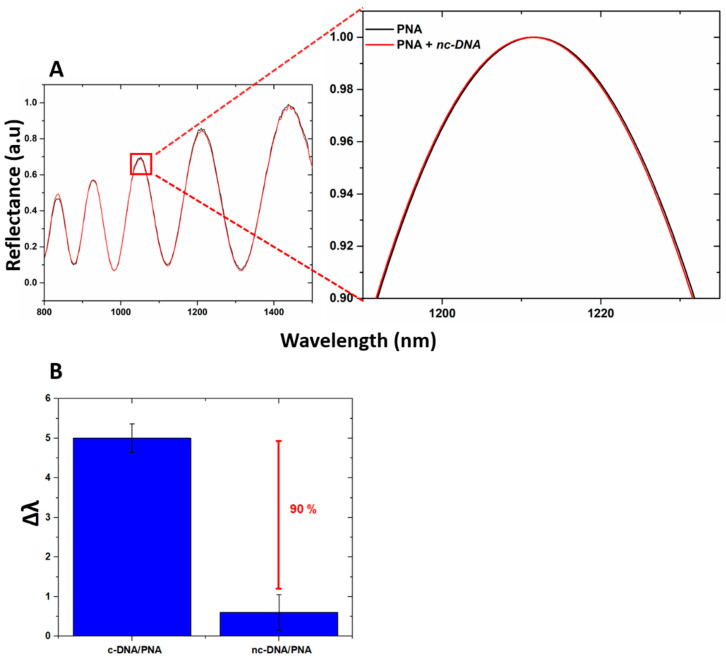
(**A**) reflectivity spectra of PNA-GO/PSi device after the DNA polyT incubation (nc-DNA); (**B**) comparison between reflectance shift values (Δλ) of the PNA-GO/PSi device after interaction with 100 µM c-DNA and 100 µM nc-DNA.

The specificity of the device for the DNA target was investigated by exposing the PSi-based biosensor to increasing concentrations of DNA polyT sequence up to 100 µM. The data obtained showed no peak shift, confirming the selectivity of the device as well as excluding the occurrence of non-specific interactions with the transducer surface (Figure 7A). Moreover, by incubating the device with c-DNA and nc-DNA (both at 100 µM) and by analyzing their reflectance, a difference between the signals of 90% was measured, as shown in Figure 7B highlighting the specificity of the device when interrogated via reflectance modality.

The interaction between PNA, immobilized on the device surface, and the DNA at different concentrations (25, 50, 75, and 100 µM, pH 5) was also investigated by PL analysis, monitoring the changes in the GO light emission. The spectra reported in Figure S5 showed only a slight variation of the PL signal after DNA incubation. The result was due to a very weak effect of PNA/DNA hybridization on the surface charge of GO, whose carboxyl groups were already engaged in the PNA molecules binding. The morphological characteristics of the transducer surface were analyzed by AFM (Figure S6A–C). The topography and the non-contact mode (NCM) phase images of bare PSi showed hillocks and voids of about 100 nm distributed on the whole surface; a partial pore clocking was observed after GO functionalization, due to the distribution of GO nano-sheet on the porous surface. After the PNA/DNA interaction, the topography, but even more the NCM phase, image of the sample revealed a crowded device surface, since the organic matter (i.e., the PNA/DNA heteroduplexes) was covalently bound and accumulated on it.

**Figure 8:**
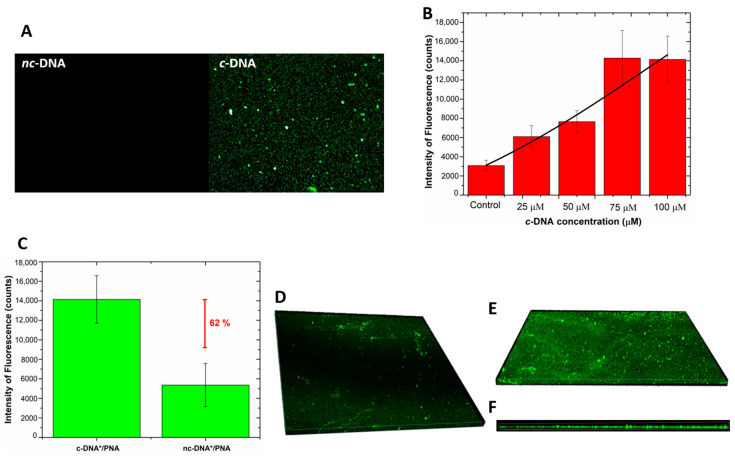
(**A**) fluorescence images of PNA-GO/PSi device incubated with fluorescent DNA (c-DNA) and DNA polyT (nc-DNA) at magnification 50×; (**B**) fluorescence intensity as a function of c-DNA concentration. The values were calculated by averaging different images of the same sample. Data were fitted by using OriginLab^TM^ Dose–response non-linear curve fit; (**C**) comparison between fluorescence intensity values of PNA-GO/PSi device after the interaction with 100 µM of fluorescent c-DNA (c-DNA*) and 100 µM of fluorescent nc-DNA (nc-DNA*); (**D**) top view of the fluorescent DNA infiltrated in the GO/PSi device (negative control); (**E**) top view and (**F**) side view of PNA/DNA infiltrated into PSi.

Fluorescence microscopy was used as a further technique to monitor the interaction of the hybrid device with labeled DNA-FAM and DNA polyT-FAM. No fluorescent signal was detected after the incubation of the device with the DNA polyT, thus completely excluding the occurrence of non-specific interactions of non-complementary DNA with the bioprobe and/or with the surface of the GO/PSi. A fluorescent signal was instead recorded in the sample treated with the complementary sequence (Figure 8A). Moreover, an increasing fluorescence trend was observed as a function of the DNA concentration; Figure 8B reports the mean fluorescence intensity of the device after interaction with the DNA for different concentrations; the values were calculated by analysis of the fluorescence images with the NIH ImageJ software [52] (data not shown here). An LOD of 18 ± 3 µM was achieved, by considering a blank intensity of 3075 ± 570 counts and the sensitivity of 96 ± 14 counts µM^−1^ (calculating in the linear range, data not shown here) of the dose–response curve (R^2^ = 0.97). Moreover, Figure 8C shows the value of the mean fluorescence intensity, calculated from fluorescence images of the device incubated with labeled c-DNA and nc-DNA (both at 100 µM) and measuring a difference between the signals of 62%, confirming the specificity of the platform.

Confocal microscopy was used to get a deep insight of the DNA infiltration into the pores after the hybridization. Figure 8D,E report the 3D representation of all focal planes of a negative control sample and a PNA-GO/PSi one recorded by the instrument. The negative control, obtained by infiltration of the labelled DNA into the GO/PSi device (without the PNA probe), showed a negligible fluorescence related to the aspecific adsorption. On the contrary, Figure 8E clearly proved the hybridization between the PNA-modified device and the complementary target at pH 5.0. Moreover, the side view represented another clear picture of the DNA infiltrated into the porous channels (Figure 8F).

## 4. Conclusions

The detection of specific DNA sequences is a powerful tool for the diagnosis and clinical analysis due to its inherent molecular property. The use of nucleic acid analogue probes in biosensor development represents an exciting area for gene-sensor technology. The PNA bioprobes, more than the usual DNA ones, are able to selectively and sensibly bind target sequences, avoiding the problem of electric charge accumulation that could let to sensor substrate degradation. The incorporation of PNA probes into nanostructured materials could thus greatly improve the detection performances. In the present work, the fabrication and characterization of a PNA-based GO/PSi device as a proof-of-concept biosensor for label-free optical detection of Brugada syndrome (BS) has been reported. The hybrid GO/PSi transducer was obtained by covalently bound the GO in the PSi sponge-like structure, previously stabilized and functionalized by a hydrosilylation process. A peculiar PNA sequence, able to specifically recognize the DNA target sequence relative to the BS, was used as a bioprobe and covalently immobilized on the device. The interaction between the synthesized PNA and the DNA target through heteroduplexes formation has been investigated in solution by CD, CD melting, and PAGE analyses. All the steps required for the fabrication and functionalization of the hybrid optical device, as well as bioprobe conjugation, have been monitored by a label-free optical method based on spectroscopic reflectometry/PL and by imaging analysis based on SEM/AFM. Reflectivity analysis, fluorescence, and confocal microscopy were used to quantify the hybridization process on the hybrid chip made of GO/PSi. The PNA probe, even bound to the nanostructured surface, recognized the DNA from the non-complementary one at different concentrations up to 100 µM, showing an LOD of 25 ± 2 µM, through the use of the reflectivity analysis, and an LOD of 18 ± 3 µM, by using fluorescence. Moreover, a higher specificity of the PNA-GO/PSi device has been confirmed by the reflectivity analysis with respect to the fluorescent modality. The fabrication and measurement procedures reported in this work endorsed the hybrid GO/PSi support as a suitable transducer for the development of a PNA biosensor for non-invasive analyses of a molecular target. Moreover, the label-free optical PNA based GO/PSi sensor, easy to use, with fast response time, high sensibility and specificity, represents a good starting point for the realization of portable devices acts to replace costly and time-consuming analysis typical of genetic tests. The capability of the biosensor to discriminate between the target and non-target sequences was evaluated by using “clean” samples. Therefore, it is important to stress that, despite the excellent results obtained, many other efforts still need to be made to allow the detection of BS from “real samples”. The optimization of the surface stabilization as well as the tailoring of the pore size of the porous matrix might be a starting point for avoiding non-specific attachment of biomolecules on the PSi device.

## Figures and Tables

**Table 1:** Complementary (DNA-FAM and DNA) and not-complementary (DNA polyT-FAM and DNA polyT) DNA target and PNA sequences synthetized by solid-phase strategy.

Sample	Sequence
DNA-FAM	6FAM-AGGAGAGCACCGAGCCCCTGAG (5′-3′)
DNA	AGGAGAGCACCGAGCCCCTGAG (5′-3′)
DNA polyT-FAM	6FAM-CCTTTTTTTTTT (5′-3′)
DNA polyT	CCTTTTTTTTTT (5′-3′)
PNA	AcNH-gggctcggtgctKK-NH_2_ (N to C)

## Supplementary Materials

Supplementary data related to this article are available online at https://www.mdpi.com/2079-4991/10/11/2233/s1. Figure S1. (A) ESI-MS spectrum of PNA recorded in the positive ion mode. Calcd. for [M + 2H]^2+^ 1807.74, found 1807.7, calcd. for [M + 3H]^3+^ 1205.49, found 1205.5; calcd. for [M + 4H]^4+^ 904.37, found 904.3. (B) Isotopic peaks distribution of [M + 2H]^2+^ pseudomolecular ion. (C) Isotopic peaks distribution of [M + 4H]^4+^ pseudomolecular ion. Figure S2. SEM images of the top and side view of PSi before (A) and after GO infiltration (B). Figure S3. CD spectra of DNA and DNA FAM alone (black solid line, panels A and B, respectively) and after annealing with PNA (dashed line A and B). CD profiles of the arithmetic sum of DNA or DNA FAM and PNA are reported as red lines, the CD spectrum of PNA is reported as a dotted line. All samples were dissolved in 100 mM PBS. CD profiles were normalized at 320 nm; Table (C) λ values for CD minima and maxima of each sample. Figure S4. (A) Overlapped CD melting profiles of DNA FAM/PNA (green line) and DNA/PNA (blue line) mixtures at 1:0.8 ratio. The curves were obtained by monitoring the absorbance at 269 nm for DNA FAM/PNA and 268 nm for DNA/PNA at a heating rate of 1 °C/min; (B) Table with Tm values of each sample. Figure S5. Photoluminescence spectra of PSi-PNA after DNA hybridization. Figure S6. Topography and NCM phase images of bare-PSi (A), GO/PSi (B) and PNA-GO/PSi after DNA hybridization (C). Figure S7. Not processed image of PAGE analysis.
