# Supplementary material for: PNA-Based Graphene Oxide/Porous Silicon Hybrid Biosensor: Towards a Label-Free Optical Assay for Brugada Syndrome
Source: Nanomaterials (Basel). 2020 Nov 10;10(11):2233. doi: 10.3390/nano10112233 (PMC7697640; doi:10.3390/nano10112233)
Supplement: Supplementary file 1 [file nanomaterials-10-02233-s001.pdf]

nanomaterials

Supplementary Materials

PNA-Based Graphene Oxide/Porous Silicon Hybrid Biosensor: Towards a Label-Free Optical Assay for Brugada Syndrome

Rosalba Moretta [1], Monica Terracciano [2,*], Nicola Borbone [2], Giorgia Oliviero [3], Chiara Schiattarella [4], Gennaro Piccialli [2], Andrea Patrizia Falanga [3], Maria Marzano [5], Principia Dardano [1], Luca De Stefano [1,*], and Ilaria Rea [1]

1 Institute of Applied Sciences and Intelligent Systems, National Research Council, 80131, Naples, Italy; rosalba.moretta@na.isasi.cnr.it (R.M.); principia.dardano@na.isasi.cnr.it (P.D.); ilaria.rea@na.isasi.cnr.it (I.R.)
2 Department of Pharmacy, University of Naples Federico II, 80131, Naples, Italy; nicola.borbone@unina.it (N.B.); gennaro.piccialli@unina.it (G.P.)
3 Department of Molecular Medicine and Medical Biotechnologies, University of Naples Federico II, 80131, Naples, Italy; giorgia.oliviero@unina.it (G.O.); andreapatrizia.falanga@unina.it (A.P.F.)
4 Department of Physics "E. Pancini", University of Naples Federico II, 80126 Naples, Italy; ch.schiattarella@gmail.com (C.S)
5 Institute of Crystallography, National Research Council, 70126, Bari, Italy; maria.marzano@unina.it (M.M)
* Correspondence: monica.terracciano@unina.it (M.T.); luca.destefano@na.isasi.cnr.it (L.D.S.); Tel.: +39-081-678521 (M.T.); +39-081-6132594 (L.D.S.)

A

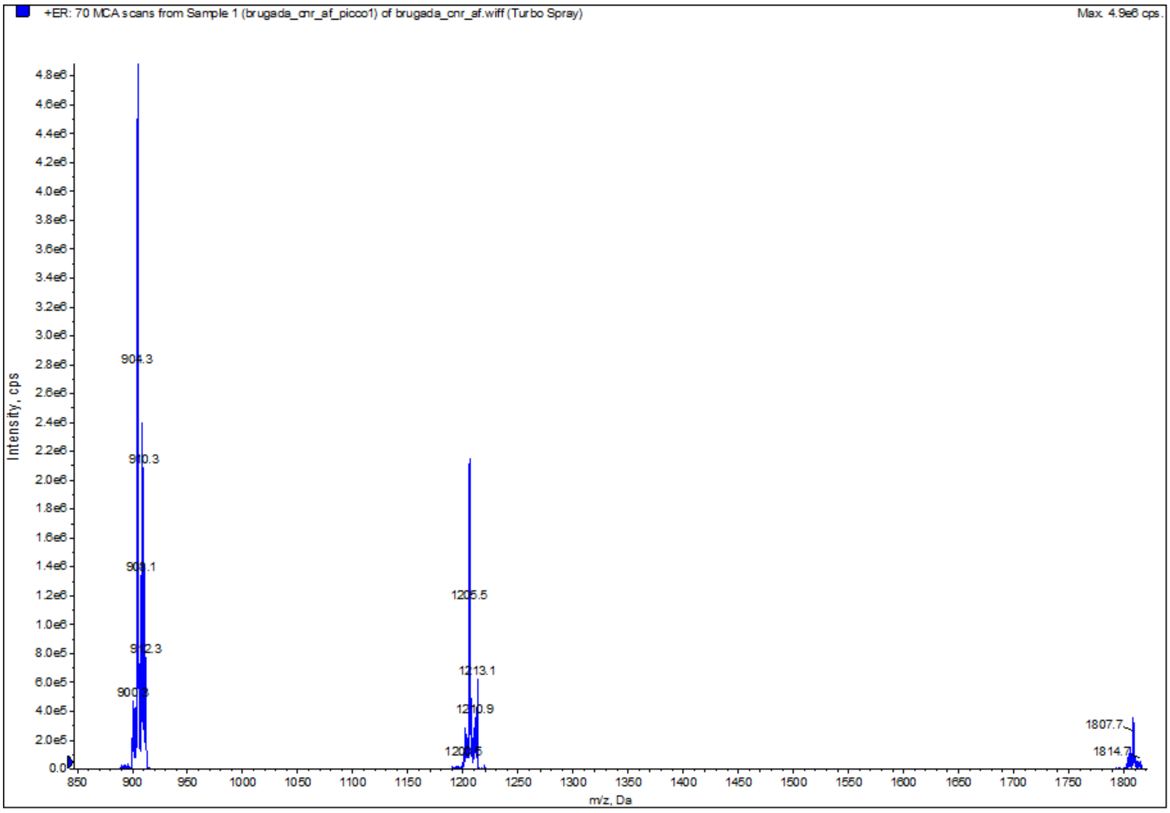
+ER: 70 MCA scans from Sample 1 (brugada_cnr_af_picco1) of brugada_cnr_af.wiff (Turbo Spray)
Max. 4.9e6 cps.
Intensity, cps
m/z, Da
1807.7
1814.7

B

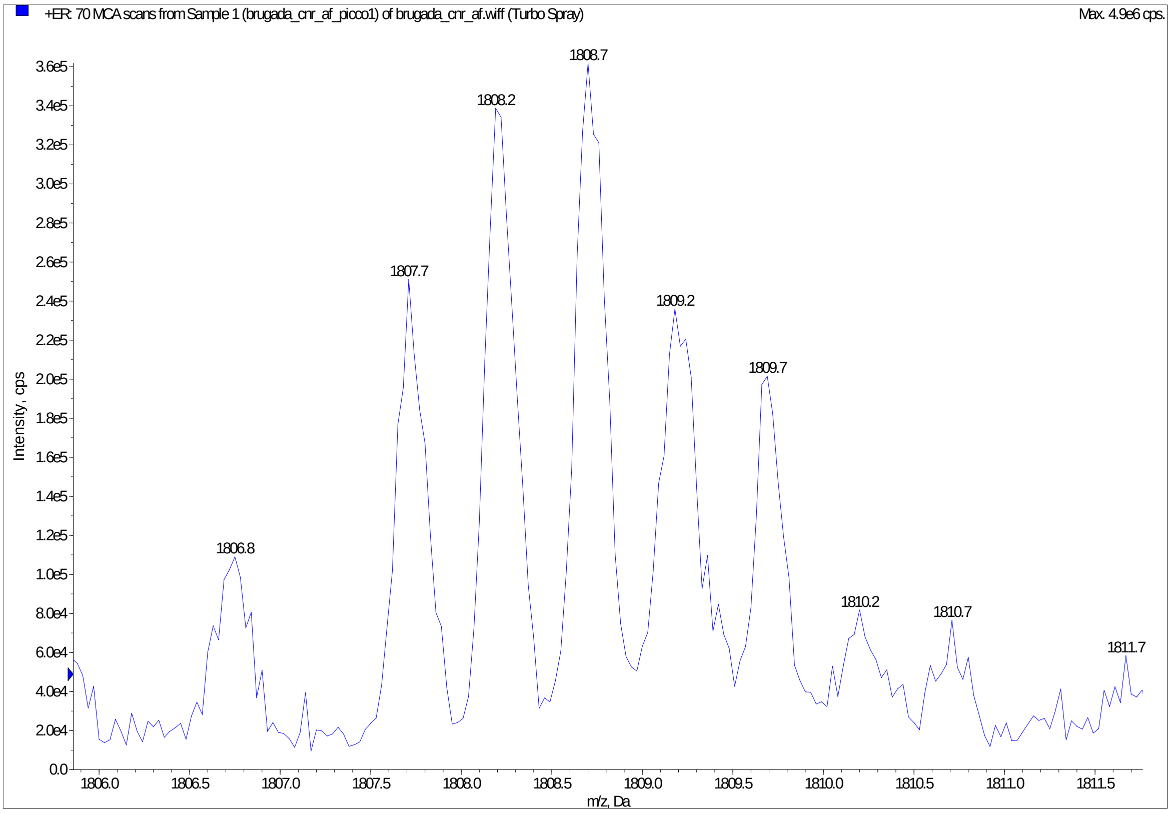
+ER: 70 MCA scans from Sample 1 (brugada_cnr_af_picco1) of brugada_cnr_af.wiff (Turbo Spray)
Max. 4.9e6 cps.
Intensity, cps
m/z, Da
1806.8
1807.7
1808.2
1808.7
1809.2
1809.7
1810.2
1810.7
1811.7

C

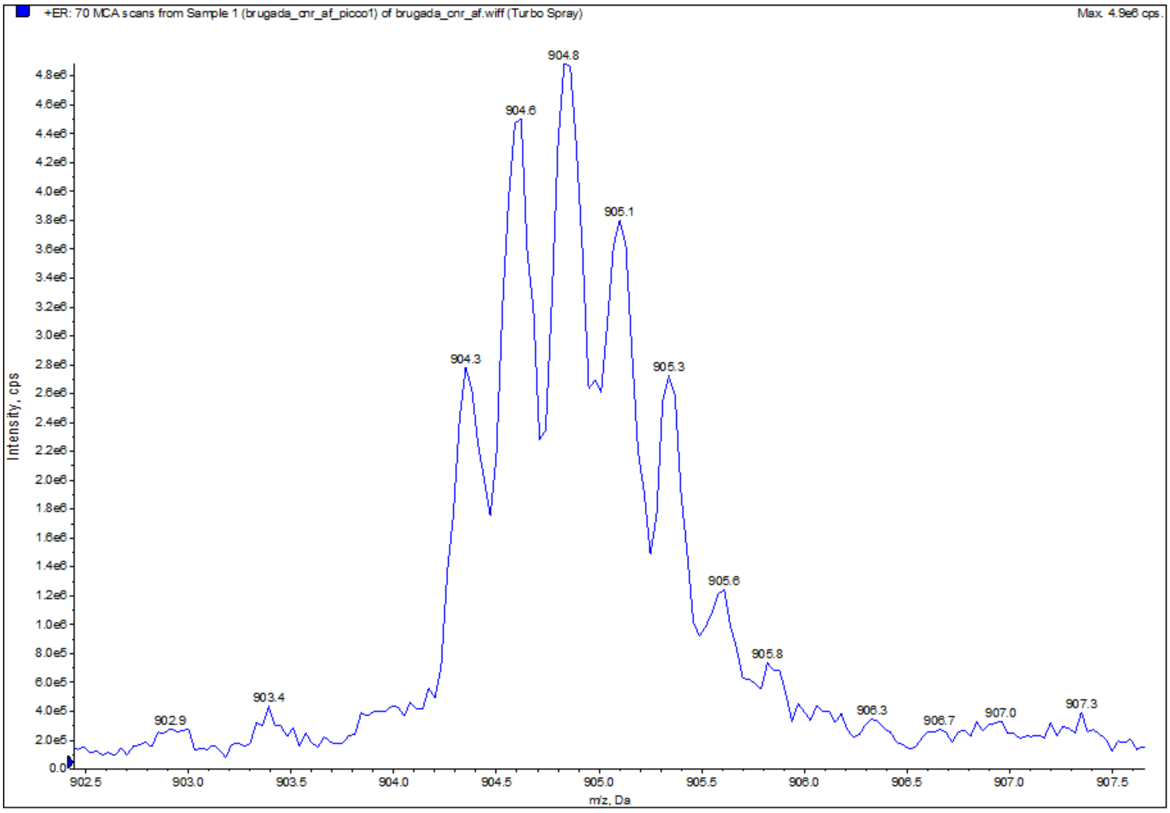

Figure S1. (A) ESI-MS spectrum of PNA recorded in the positive ion mode. Calcd. for $[M + 2H]^{2+}$ 1807.74, found 1807.7, calcd. for $[M + 3H]^{3+}$ 1205.49, found 1205.5; calcd. for $[M + 4H]^{4+}$ 904.37, found 904.3. **(B)** Isotopic peaks distribution of $[M + 2H]^{2+}$ pseudomolecular ion. **(C)** Isotopic peaks distribution of $[M + 4H]^{4+}$ pseudomolecular ion.

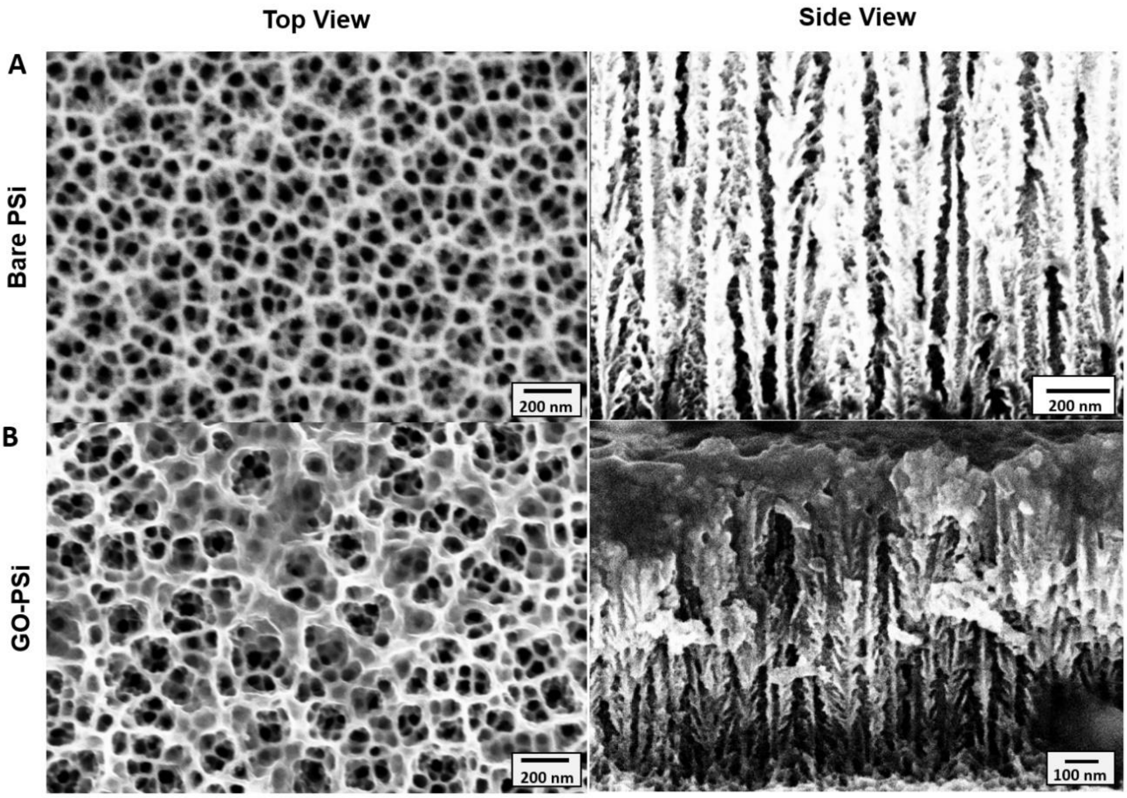

Figure S2. SEM images of the top and side view of PSi before (**A**) and after GO infiltration (**B**).

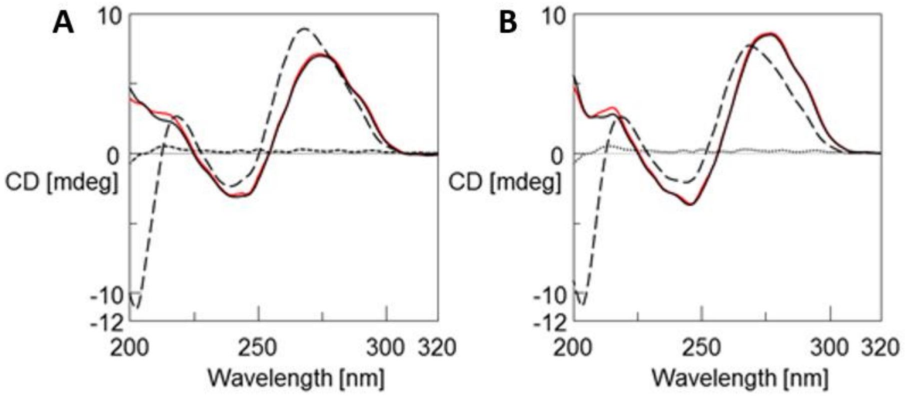

C

Sample	λ min (nm)	λ max (nm)
DNA FAM	245	215-277-290
DNA FAM/PNA	203-244	219-269
DNA	242	217-275-290
DNA/PNA	203-239	218-268

Figure S3. CD spectra of DNA and DNA FAM alone (black solid line, panel **A** and **B** respectively) and after annealing with PNA (dashed line **A** and **B**). CD profiles of the arithmetic sum of DNA or DNA FAM and PNA are reported as red lines, the CD spectrum of PNA is reported as dotted line. All samples were dissolved in 100 mM PBS. CD profiles were normalized at 320 nm; Table (**C**) λ values for CD minima and maxima of each sample.

The CD spectra of DNA FAM/PNA and DNA/PNA mixtures were different from that of the arithmetic sum of the separate components, thus confirming that the interaction between both DNA FAM and DNA oligonucleotides (ONs) with PNA occurred (Figure S3 panel A and B, respectively).

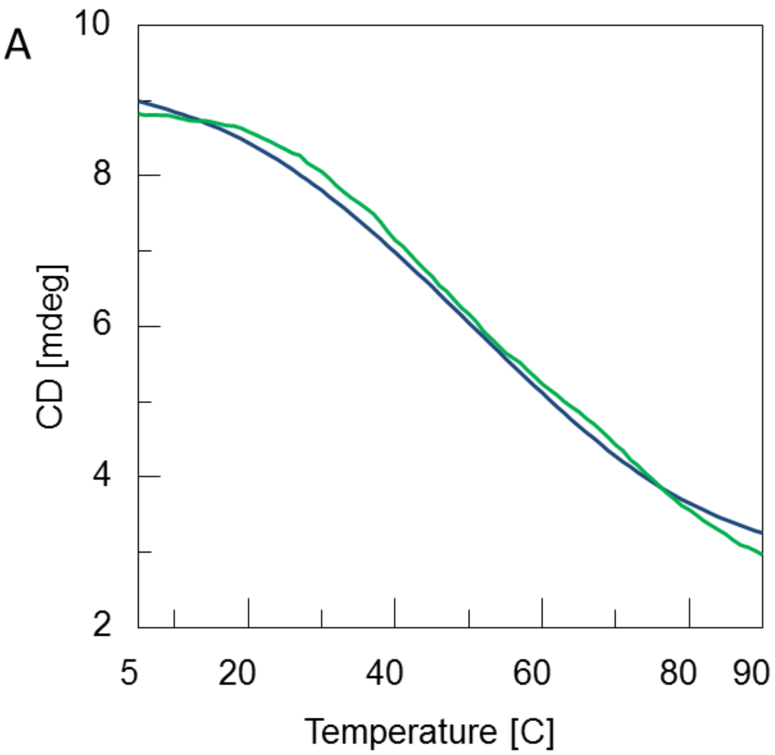

B

Samples	Tm °C
DNAwt FAM/PNA	44
DNAwt /PNA	50

Figure S4. (**A**) Overlapped CD melting profiles of DNA FAM/PNA (green line) and DNA/PNA (blue line) mixtures at 1:0.8 ratio. The curves were obtained by monitoring the absorbance at 269 nm for DNA FAM/PNA and 268 nm for DNA/PNA at a heating rate of 1 °C/min; (**B**) Table with Tm values of each sample.

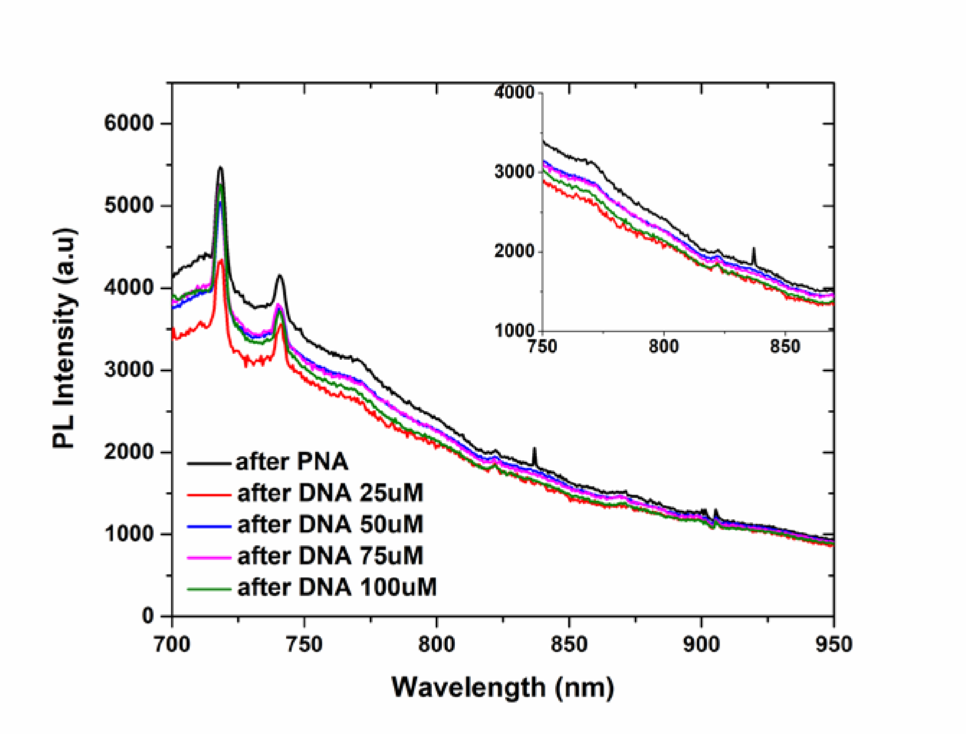

Figure S5. Photoluminescence spectra of PSi-PNA after DNA hybridization.

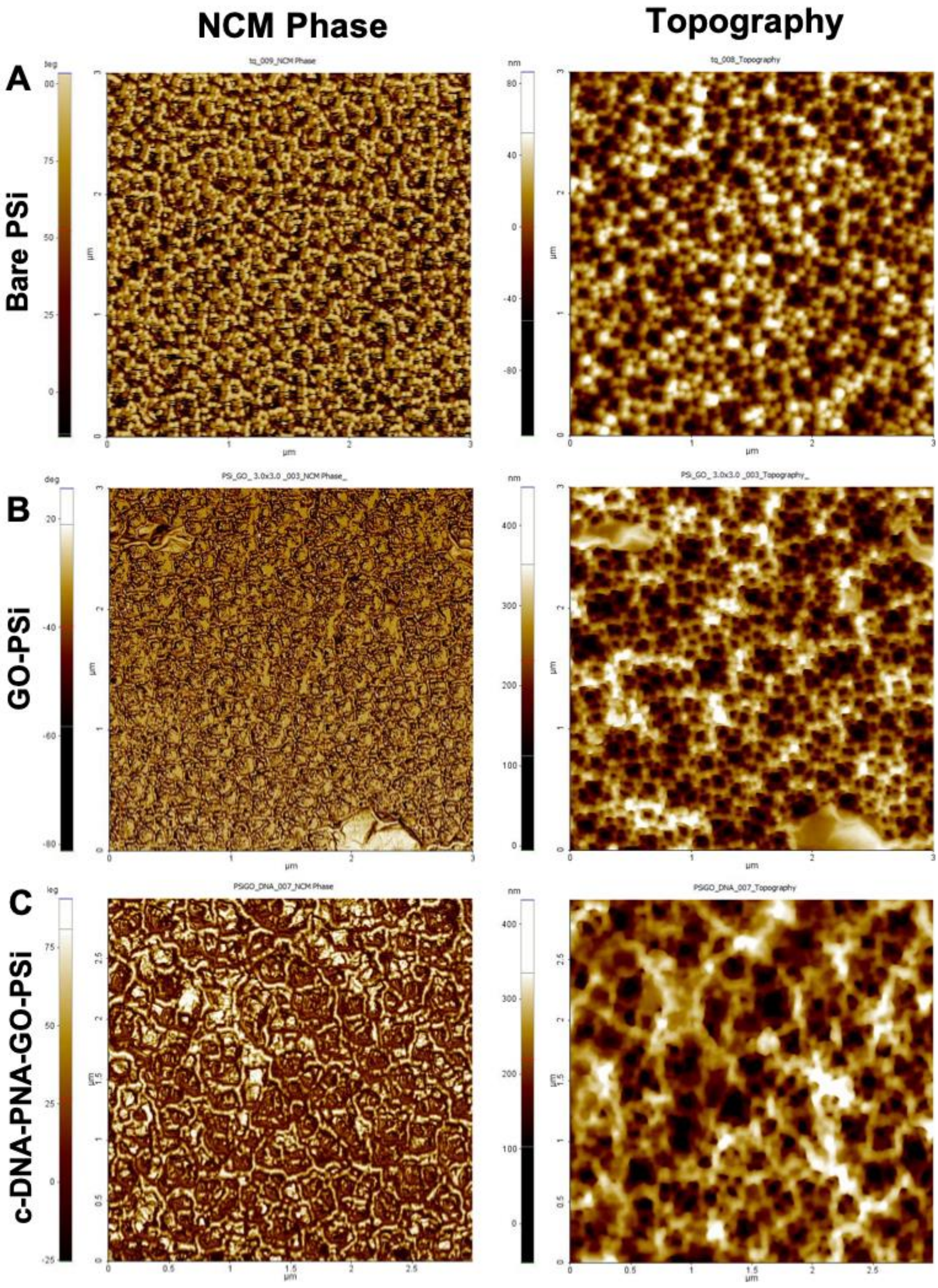

Figure S6. Topography and NCM phase images of bare-PSi (**A**), GO/PSi (**B**) and PNA-GO/PSi after DNA hybridization (**C**).

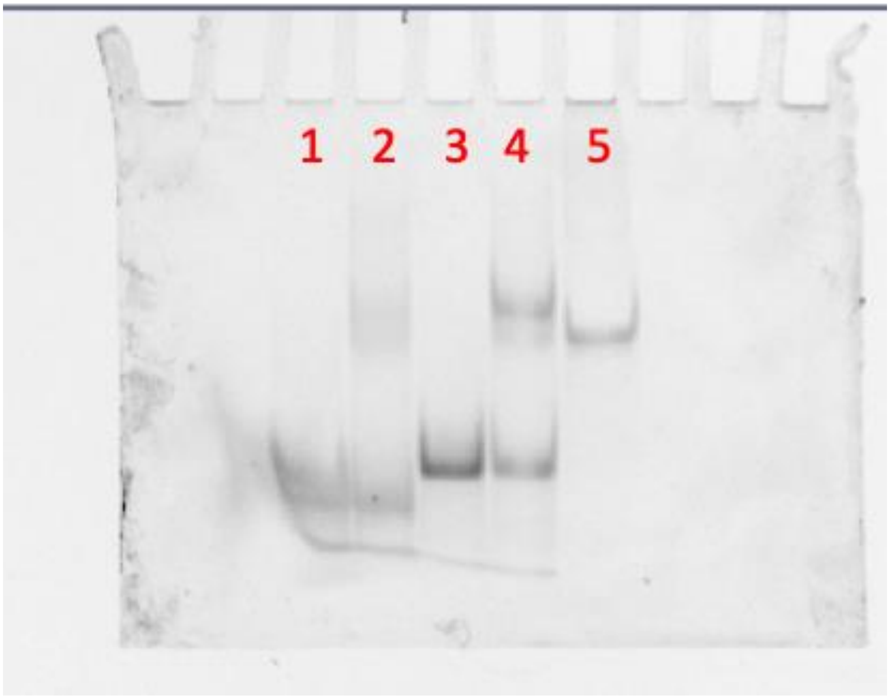

Figure S7. Not processed image of PAGE analysis.

© 2020 by the authors. Licensee MDPI, Basel, Switzerland. This article is an open access article distributed under the terms and conditions of the Creative Commons Attribution (CC BY) license (http://creativecommons.org/licenses/by/4.0/).
